# Several endocannabinoids and amino acids may be biosynthetically converted to catecholamines *in vivo*

**DOI:** 10.3389/fphar.2025.1614460

**Published:** 2025-07-18

**Authors:** Paul J. Fitzgerald

**Affiliations:** Independent Researcher, West Lafayette, IN, United States

**Keywords:** norepinephrine, noradrenaline, epinephrine, adrenaline, dopamine, stable isotope, liquid chromatography, mass spectrometry

**FIGURE 1:**
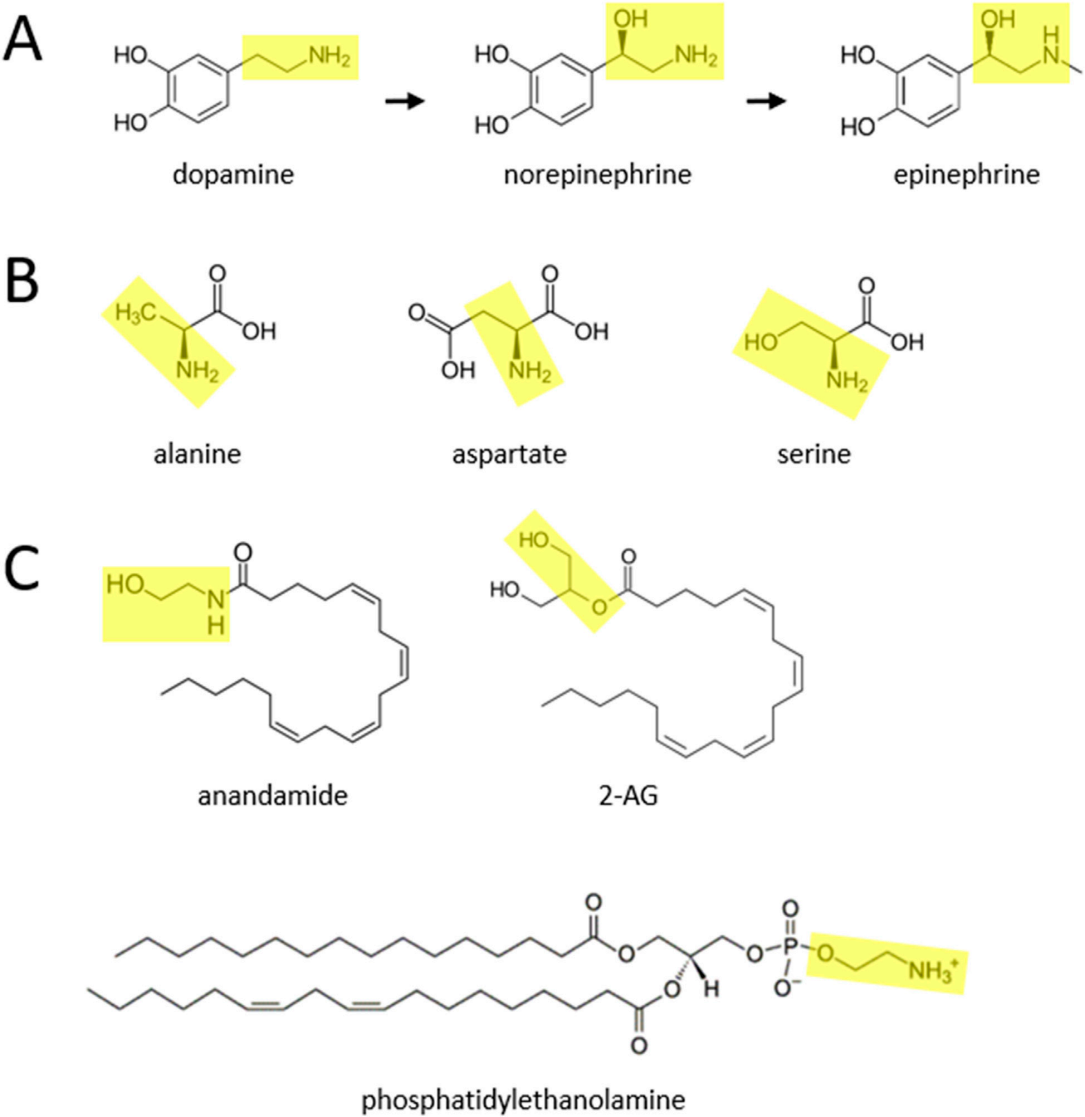
Proposed substrates for catecholamine biosynthesis. **(A)** Canonical pathway for catecholamine synthesis, described over 70 years ago, in which the amino acid tyrosine (not shown) is transformed to dopamine, and subsequently norepinephrine and epinephrine. **(B)** It is proposed here that the amino acids alanine, aspartate, and serine participate in additional pathways for synthesizing catecholamines. **(C)** It is further suggested here that the endocannabinoids anandamide and 2-AG, and lipids such as phosphatidylethanolamine, also participate in novel biosynthesis pathways. The yellow boxes highlight two-carbon moieties, with attached hydroxyl or amine groups, that are proposed to detach and react with currently unidentified benzene-ring containing (i.e., phenylic) molecules to form the side chain of catecholamines.

There is increasing interest in the topic of novel biosynthetic pathways for producing catecholaminergic neurotransmitters (dopamine, norepinephrine, epinephrine) (Fitzgerald, 2022; Wlaź et al., 2025), in addition to the single canonical pathway described over 70 years ago that uses the amino acid tyrosine as a precursor to dopamine (Figure 1A) (Holtz, 1939; Blaschko, 1942; Bulbring, 1949). While there are very limited data at this time on the topic of novel pathways for catecholamine synthesis from substrates such as ethanol, it has been shown that fungi (*Aspergillus* species) can use ethanol molecules to synthesize aflatoxin, which is a small molecule toxin that can be found on certain farming plants (Furukawa et al., 2023). It has previously been suggested that a range of common dietary factors, including foods that contain the lipid phosphatidylethanolamine and the amino acid serine, may be acutely transformed to various neurotransmitters in the body (Fitzgerald, 2020). The current publication briefly examines some additional molecules, including the endocannabinoids anandamide and 2-arachidonoylglycerol (2-AG), as well as the amino acids alanine and aspartate, that may also be converted to catecholamines *in vivo*. Such biochemical transformations may have evolved to endow the body with greater flexibility and redundancy in producing these prominent catecholaminergic signaling molecules, and may also be related to the rewarding properties of endocannabinoids. For example, endocannabinoid signaling is closely associated with dopaminergic signaling in the nucleus accumbens (Kibret et al., 2023).

Alanine (MilliporeSigma; D,L-alanine-2,3,3,3-d4; catalog # 488917) and aspartate (MilliporeSigma; D,L-aspartic acid-2,3,3-d3; catalog # 589667) are non-essential amino acids that are nonetheless present in a variety of dietary sources. It is suggested here that these two molecules may function similarly to serine (MilliporeSigma; D,L-serine-2,3,3-d3; catalog # 688436), in providing the two-hydrocarbon chain with a terminal amine group (ethylamine), to be added to the benzene ring (i.e., phenylic) structure of an unidentified molecule or molecules, to form dopamine. (As noted in a previous publication, several phenylic candidates are the plant-based molecules catechol (MilliporeSigma; 1,2-dihydroxybenzene-d4; catalog # 795879), protocatechuic acid (MedChemExpress; protocatechuic acid-d3; catalog # HY-N0294S), catechin (MilliporeSigma; (±)-catechin-2,3,4-^13^C3; catalog # 719579), and quercetin (MedChemExpress; quercetin-d3; catalog # HY-18085S1) (Fitzgerald, 2020).) The ethylamine moiety is highlighted in yellow in Figure 1B (and also in Figure 1A, for the catecholamines), for alanine and aspartate. It is also proposed here that, as suggested in a previous publication for serine (Fitzgerald, 2020), alanine and aspartate can be decarboxylated in the process of forming dopamine. That previous publication hypothesized that the hydroxyl group of this moiety in serine can be removed to help yield dopamine, but another possibility is that the hydroxyl group remains intact and the yellow moiety within serine (ethanolamine; after decarboxylation at some point) is instead used to form norepinephrine. An additional possibility is that the amino acid cysteine, which can be synthesized endogenously from serine, can be biotransformed to catecholamines. At this time, the enzymes mediating these putative reactions have not been specified, although one possibility is that some of these are known enzymes that have these additional physiological roles.

This publication also suggests that portions of two endocannabinoids, anandamide and 2-AG (MedChemExpress; 2-arachidonoylglycerol-d5; catalog # HY-W011051S1), may also be converted to dopamine, norepinephrine, and epinephrine in the body. Anandamide contains a moiety, shown in yellow (Figure 1C), that is similar to that within serine. This moiety, if its hydroxyl group is removed, could be transferred to an as yet unidentified phenylic molecule to help form dopamine. If the hydroxyl group is preserved, it could instead lead to formation of norepinephrine. 2-AG contains a somewhat different moiety from anandamide, marked in yellow in Figure 1C. In this scenario, the hydroxyl group at either end of the 2-AG moiety (formed after enzymatic separation from the rest of the molecule) could be converted to an amine, and then in principle the moiety could be used to form either dopamine or norepinephrine, in a similar fashion to anandamide.

Anandamide belongs to a family of molecules called *N*-acylethanolamines (BOC Sciences, Shirley, NY), that are found in various plants and have a range of physiological functions, such as pain and cardiovascular regulation, that are being investigated scientifically (Gertsch, 2008). All of these molecules may be candidates for transferring their ethanolamine moiety to a yet to be identified phenylic compound or compounds to form dopamine (or norepinephrine), as was hypothesized in a previous publication for the lipid phosphatidylethanolamine (Figure 1C) (Fitzgerald, 2020). A potentially related point is that, in a range of organisms, the enzyme fatty acid amide hydrolase (FAAH) is known to break down anandamide into ethanolamine (Maccarrone et al., 1999). Phosphatidylethanolamine, we suggest here, may also be involved in the (reversible) biosynthesis of triglycerides (also known as triacylglycerols) (Horvath et al., 2011), thereby potentially linking this molecule with the deleterious health-related effects of triglycerides. Two other lipids, phosphatidylcholine and phosphatidylserine (BOC Sciences, Shirley, NY), could also be transformed to catecholamines. To do so, phosphatidylcholine may need to have its three methyl groups enzymatically removed from its terminal moiety. Phosphatidylserine, in contrast, may function much like serine itself, in that its serine group could be converted to either dopamine or norepinephrine as described above. Phosphatidylserine can also be transformed to phosphatidylethanolamine in bacteria (Kanfer and Kennedy, 1963).

Consistent with the hypothesis that portions of endocannabinoid molecules can be biotransformed to dopamine and norepinephrine *in vivo*, there is a significant literature in animals linking endocannabinoid signaling with that in catecholamines. For example, dopamine D2 receptor activation interacts with endocannabinoid-mediated long-term depression (LTD) in midbrain dopamine neurons (Pan et al., 2008). Given the role of norepinephrine in memory consolidation and synaptic plasticity in general, it is interesting to note that cannabidiol, which interacts with 2-AG and anandamide, can reverse hippocampal long-term potentiation (Hughes and Herron, 2019). Further studies also implicate cannabidiol in hippocampal synaptic plasticity, possibly through interaction with endocannabinoids (Castelli et al., 2023). While these studies do not directly link endocannabinoids with catecholamine synthesis, they do highlight extensive interaction between these different signaling pathways.

In a similar manner to the description in previous publications (Fitzgerald, 2021; Wlaź et al., 2025), the hypothesis put forth here that several endocannabinoids and amino acids are biotransformed to catecholamines can be tested using stable isotope biochemistry followed by liquid chromatography-mass spectrometry. In such an experiment, one group of the model organisms (such as mice or rats) should be administered “heavy” (carbon 13 or deuterium) molecules, whereas other animals would be a control group that gets the unlabeled molecule (carbon 12 or normal hydrogen). A second control group could get only a saline injection. (Catalog numbers are listed above for commercially available stable isotope versions of many of the molecules described in this publication.) The stable isotope labeling should include one or more atoms within the yellow moieties shown in Figure 1. The stable isotope versions of the three amino acids described above perhaps could be systemically administered (i.e., intraperitoneally, subcutaneously, or intravenously), whereas stable isotope endocannabinoids should perhaps be directly infused into the brain (so they are not immediately degraded in the periphery), where the subsequent measurement of labeled dopamine, norepinephrine, and epinephrine could be coupled with microdialysis, especially in the case of endocannabinoids. Further experimentation could help evaluate the overall hypothesis proposed here, by examining whether dietary tyrosine depletion in rodents or pharmacological inhibition of tyrosine hydroxylase with the drug AMPT, markedly inhibits catecholamine synthesis due to canonical pathway blockade. Additional experiments could be carried out in dopamine beta hydroxylase knockout mice, which lack canonical norepinephrine synthesis, to examine whether catecholamine synthesis nonetheless occurs.
